# PD-1 inhibitor combined with paclitaxel and cisplatin in the treatment of recurrent and metastatic hypopharyngeal/laryngeal squamous cell carcinoma: efficacy and survival outcomes

**DOI:** 10.3389/fimmu.2024.1353435

**Published:** 2024-05-17

**Authors:** Qi Fang, Xiaodi Li, Pengfei Xu, Fei Cao, Di Wu, Xinrui Zhang, Chunyan Chen, Jianming Gao, Yong Su, Xuekui Liu

**Affiliations:** ^1^ Department of Head and Neck Surgery, Sun Yat-Sen University Cancer Center, Guangzhou, China; ^2^ State Key Laboratory of Oncology in South China, Guangdong Provincial Clinical Research Center for Cancer, Sun Yat-sen University Cancer Center, Guangzhou, China; ^3^ Collaborative Innovation Center for Cancer Medicine, Guangzhou, Guangdong, China; ^4^ Department of Otolaryngology, the Fifth Affiliated Hospital of Guangzhou Medical University, Guangzhou, China

**Keywords:** immunotherapy, PD-1 inhibitors, recurrence and metastasis, hypopharyngeal squamous cell carcinoma, laryngeal squamous cell carcinoma immunotherapy, laryngeal squamous cell carcinoma

## Abstract

**Objective:**

This retrospective study analyzed the efficacy of PD-1 inhibitors combined with albumin-bound paclitaxel and cisplatin (TP regimen) in the treatment of recurrent and metastatic hypopharyngeal/laryngeal squamous cell carcinoma (RMHSCC/RMLSCC).

**Methods:**

Patients diagnosed and treated at the Sun Yat-sen University Cancer Center from August 1, 2020, to August 15, 2023, with histologically confirmed RMHSCC/RMLSCC were included. All patients received PD-1 inhibitors combined with albumin-bound paclitaxel (260mg/m2) and cisplatin (60mg/m2) for 3–4 cycles. The primary endpoints were overall survival (OS) and progression-free survival (PFS).

**Results:**

A total of 50 patients with RMHSCC/RMLSCC who received TP+PD-1 inhibitor therapy were included, with an objective response rate (ORR) of 56.0% (28/50). The 1-year and 2-year OS rates were 80.2% (95% CI: 69.3%-92.9%) and 68.6% (95% CI: 52.6%-89.5%), respectively, while the 1-year and 2-year PFS rates were 44.7% (95% CI: 31.9%-62.5%) and 26.0% (95% CI: 12.6%-53.4%), respectively. Treatment-related adverse events mainly included rash, myelosuppression, gastrointestinal reactions, and hypothyroidism.

**Conclusion:**

In the treatment of RMHSCC/RMLSCC with TP + PD-1 inhibitors, survival rates of patients can be improved while ensuring the safety of the treatment regimen.

## Introduction

Head and neck squamous cell carcinoma (HNSCC) is considered the sixth most common cancer globally, causing approximately 500,000 deaths annually (1). Projections suggest that by 2030, the annual incidence may reach 1.08 million cases. Despite advancements in treatments such as surgery and comprehensive therapy, the overall 5-year survival rate still hovers between 40–50% (2). This is primarily due to high recurrence rates, aggressive growth, and propensity for metastasis. Hypopharyngeal squamous cell carcinoma and laryngeal squamous cell carcinoma, as part of HNSCC, have been relatively understudied and are often grouped together with other types of HNSCC. However, due to their unique anatomical locations, these subtypes require more targeted research and analysis (3).

Programmed death-1 (PD-1) inhibitors can block the binding of PD-1 and PD-L1, upregulate the growth and proliferation of T cells, enhance T cell recognition of tumor cells, and ultimately boost the body’s immune function to achieve antitumor effects (4). The prospective phase III randomized controlled trial CheckMate-141 (5) demonstrated excellent efficacy of the PD-1 inhibitor nivolumab monotherapy in recurrent or metastatic head and neck squamous cell carcinoma (R/M HNSCC). The Keynote-048 study (6), as a phase III clinical trial, showed that the median overall survival (OS) of R/M HNSCC treated with the PD-1 inhibitor pembrolizumab combined with chemotherapy (cisplatin + 5-fluorouracil) was higher than that of the EXTREME regimen (cetuximab + platinum and 5-fluorouracil). The Food and Drug Administration (FDA) has approved nivolumab and pembrolizumab for second-line treatment of R/M HNSCC, and pembrolizumab monotherapy and pembrolizumab combined with platinum chemotherapy have become new standards of care for R/M HNSCC (7, 8). However, there is still a lack of clinical evidence of PD-1 inhibitors combined with chemotherapy in recurrent and metastatic hypopharyngeal/laryngeal squamous cell carcinoma (RMHSCC/RMLSCC). Since 2019, our institution has conducted studies on PD-1 inhibitors combined with albumin-bound paclitaxel plus cisplatin (TP regimen) for the treatment of HNSCC, including two phase II clinical trials, NCT04826679 and NCT06130007. Our previous studies have shown that PD-1 inhibitor + TP achieved a 94.1% response rate in neoadjuvant treatment of hypopharyngeal/laryngeal cancer (9). However, there is still a lack of clinical evidence of PD-1 inhibitors combined with chemotherapy in patients with RMHSCC/RMLSCC (10). This retrospective study aims to analyze the efficacy and safety of PD-1 inhibitors combined with albumin-bound paclitaxel and cisplatin in the treatment of recurrent or metastatic hypopharyngeal/laryngeal squamous cell carcinoma.

## Methods

### Study design

This experiment was a single-center, retrospective study. Recruitment of Patients with RMLSCC/RMHSCC meeting the inclusion criteria and diagnosed clearly was initiated at the Sun Yat-sen University Cancer Center from August 1, 2020, to August 15, 2023. These patients were treated with 3–4 cycles of albumin-bound paclitaxel plus cisplatin (TP regimen) combined with one PD-1 inhibitor (Camrelizumab, Pembrolizumab, Sintilimab, Toripalimab, or Tislelizumab). Efficacy assessment was conducted 2 weeks after the completion of treatment with TP+PD-1 inhibitors. In cases of complete response (CR), definitive radiotherapy was administered. For patients achieving partial response (PR), those with a reduction in tumor volume of ≥70% on imaging were defined as having major PR and received concurrent chemoradiotherapy. PR patients who did not meet the criteria for major PR were defined as having minor PR and underwent definitive surgery followed by adjuvant chemoradiotherapy. Patients assessed as having stable disease (SD) or progressive disease (PD) underwent definitive surgery followed by adjuvant chemoradiotherapy. CR and PR were considered effective outcomes of treatment with TP+PD-1 inhibitors, while SD and PD were deemed ineffective. The comprehensive treatment plans for all patients were developed through multidisciplinary collaboration at the Sun Yat-sen University Cancer Center Head and Neck Department. The primary endpoints of this study were overall survival (OS) and progression-free survival (PFS). This research had been approved by the Ethics Committee of Sun Yat-sen University Cancer Center, with approval number B2023–481-01. Due to the retrospective nature of the study, informed consent from patients was waived.

Inclusion criteria: (1) Patients aged 18–70 years old; (2) Patients selected must be histologically confirmed to have squamous cell carcinoma in the hypopharynx/laryngeal region, and T and N staging confirmed through enhanced computed tomography (CT) of the head and neck and electronic fiberoptic laryngoscopy; (3) Patients with recurrent or metastatic hypopharyngeal/laryngeal squamous cell carcinoma, with distant metastases confirmed histologically as squamous cell carcinoma metastases; (4) Patients with normal major organ function, hemoglobin (Hb) ≥80 g/L, platelets (PLT) ≥100*10^9^/L, white blood cells (WBC) ≥3.5*10^9^/L, alanine aminotransferase (ALT) ≤2.5*upper limit of normal (ULN), aspartate aminotransferase (AST) ≤2.5*ULN, blood urea nitrogen (BUN) ≤1.25*ULN, creatinine (Cr) ≤1.25*ULN; (5) Selected patients require completion of enhanced CT scans of the head and neck and chest, thyroid and cervical lymph node color Doppler ultrasonography, electronic fiberoptic gastroscopy, abdominal ultrasound examination of the liver, gallbladder, pancreas, spleen, and kidneys, electrocardiogram, and other examinations; (6) Selected patients must have complete medical records.

Exclusion criteria: (1) Patients with primary hypopharyngeal/laryngeal cancer who are treatment-naïve and without distant metastases; (2) Patients with synchronous multiple primary tumors in other locations; (3) Patients with impaired major organ function who are unable to tolerate general anesthesia; (4) Patients with a history of other tumor diseases; (5) Patients with central nervous system diseases, psychiatric disorders, or other conditions leading to short-term or long-term loss of insight; (6) Patients who had previously received PD-1 inhibitor therapy.

### Treatment regimen

#### TP+PD-1 inhibitor treatment regimen

Enrolled patients underwent 3–4 cycles of TP+PD-1 inhibitor treatment at our hospital. The TP+PD-1 inhibitor 3-cycle neoadjuvant treatment regimen was as follows: On Day 1, albumin-bound paclitaxel 260mg/m^2^ and cisplatin 30mg/m^2^; On Day 2, cisplatin 30mg/m^2^ and PD-1 inhibitor 200mg (Camrelizumab, Pembrolizumab, Sintilimab, Toripalimab, or Tislelizumab) (body surface area m2 = 0.0061 * height in cm + 0.0128 * weight in kg - 0.1529). The time point for efficacy assessment was set after 3 cycles of TP+PD-1 inhibitor treatment. If CR/major PR was not achieved at this time, but the patient refused surgical treatment and all examination indicators such as blood routine, liver and kidney function, electrocardiogram, etc., were within normal range and adverse reactions were tolerable, the patient received a 4th cycle of TP+PD-1 inhibitor treatment. Evaluation was conducted again after 4 cycles of TP+PD-1 inhibitor treatment. All patients receiving TP+PD-1 inhibitor treatment during chemotherapy required prophylactic use of granulocyte colony-stimulating factor, anti-allergic, gastrointestinal protection drugs, and underwent blood routine examination every 3 days to detect bone marrow suppression.

#### Assessment of efficacy of TP+PD-1 inhibitor treatment

Two weeks after the completion of TP+PD-1 inhibitor treatment, patients underwent enhanced CT of the head and neck, electronic fiberoptic laryngoscopy, and histopathological examination to assess efficacy. Clinical neoadjuvant efficacy was assessed according to the latest version of the Response Evaluation Criteria in Solid Tumors, namely RECIST 1.1 version. Objective response rate (ORR) refers to the proportion of patients whose tumors shrink to a certain extent and maintain that size for a certain period, including cases of CR and partial response PR.

#### Follow-up treatment after TP+PD-1 inhibitor therapy

If evaluation of the primary lesion achieved CR, the patient underwent definitive radiotherapy. If PR was achieved, based on the enhanced CT results of the head and neck 2 weeks after neoadjuvant treatment, the response was classified as major PR or minor PR: Major PR was defined as a ≥70% reduction in tumor volume on enhanced CT, and concurrent chemoradiotherapy was administered; Minor PR was defined as a <70% reduction in tumor volume on enhanced CT. For patients assessed as having minor PR, SD, or PD, definitive surgery of the primary lesion was performed, followed by concurrent chemoradiotherapy within 6 weeks postoperatively. If distant metastasis was present in the patient at this time, either single-agent immunotherapy maintenance treatment or oral chemotherapy drugs were administered. Principles for radiotherapy alone and concurrent chemoradiotherapy followed the guidelines of the National Comprehensive Cancer Network (NCCN).

For patients with a history of radiotherapy who required re-irradiation, we followed the recommendations outlined in the 2018 “AHNS series: Do you know your guidelines? Guideline recommendations for recurrent and persistent head and neck cancer after primary treatment” (11) published in the Head & Neck Journal. The criteria for re-irradiation were as follows: Selection criteria include patients who are more than 6 months out from primary radiotherapy, have an ECOG of 0–2, and can receive additional therapy without exceeding a cumulative dose of 50–60 Gy to the spinal cord. Patients whose disease recurs within 6 months of radiation are typically not considered candidates for conventional reirradiation as their tumor is likely radioresistant, especially in those who received radiosensitizing chemotherapy. Other radiation strategies that can be considered in this population include altered fractionation schedules, brachytherapy, or radiosensitizing chemotherapy with a different agent than previously given (if any was given at all).

#### Assessment and treatment of neck lymph nodes

For patients with minor PR, SD, or PD after TP+PD-1 inhibitor treatment, if preoperative imaging evaluation indicates cN1 or above, selective neck lymph node dissection is performed simultaneously with surgery of the primary lesion. If postoperative pathology indicates lymph node metastasis, adjuvant radiotherapy is administered. For patients with CR, major PR, or those who did not undergo neck lymph node surgery, neck enhanced CT and neck lymph node color Doppler ultrasound are performed 2 months after the completion of radiotherapy. Compared with pre-neoadjuvant treatment enhanced CT, if suspicious abnormal lymph nodes are detected, positron emission tomography-computed tomography (PET-CT) and ultrasound-guided needle biopsy are conducted. If PET-CT shows high metabolic activity in neck lymph nodes, or needle biopsy reveals neck lymph node metastasis, neck lymph node dissection is performed.

### Follow-up and data analysis

#### Follow-up

According to the National Comprehensive Cancer Network (NCCN) guidelines for head and neck tumors, the follow-up plan for this study is as follows: In the first 2 years after treatment completion, patients need to undergo imaging examinations every 2 to 4 months. From the 3rd to the 5th year after treatment completion, the follow-up frequency is adjusted to every 3 to 6 months. Five years after treatment completion, the follow-up frequency is further reduced to once a year. Patients are followed up if there are any changes in their condition. Follow-up examinations include enhanced CT of the head and neck and chest, thyroid and cervical lymph node color Doppler ultrasound, electronic fiberoptic laryngoscopy, and if necessary, electronic fiberoptic gastroscopy. Patients at high risk of distant metastasis undergo PET-CT or whole-body bone scintigraphy. Patients who received radiotherapy and chemotherapy need to undergo blood routine, biochemical, and endocrine (thyroid and pituitary) examinations. Follow-up information is mainly recorded through outpatient follow-up records, and follow-up is conducted through methods such as telephone calls, messages, and hospital rechecks. Data on adverse events and laboratory abnormalities were collected regularly throughout treatment and for 30 days thereafter (90 days for serious adverse events and events of interest) and graded according to the National Cancer Institute Common Terminology Criteria for Adverse Events (version 4.0).

#### Data collection

Patient demographic information such as gender, age, anatomical subsites of hypopharyngeal/laryngeal cancer (pyriform sinus, postcricoid area, posterior pharyngeal wall, vocal cords, epiglottis, etc.), TNM staging (according to AJCC guidelines), tumor differentiation degree (low, moderate, high differentiation), smoking history, alcohol consumption history, treatment details including efficacy of TP+PD-1 inhibitor treatment (CR, major PR, minor PR, SD, PD), subsequent treatment regimens after TP+PD-1 inhibitor treatment, past treatment history, and follow-up information such as follow-up dates, mortality, metastasis, recurrence, specific sites of recurrence or metastasis, mental status, etc., are collected through outpatient or inpatient medical records systems, telephone communications, and other follow-up methods.

#### Statistical analysis

PFS refers to the time from the end of treatment to disease progression or the last follow-up. OS refers to the time from the end of treatment to patient death or the last follow-up. Descriptive statistics for continuous data are presented as mean ± standard deviation (Mean ± SD), while categorical variables are presented as frequencies and corresponding percentages. Data analysis was performed using SPSS 21.0 software. Differences between categorical data were assessed using the chi-square test, and risk factors for survival analysis were explored using univariate and multivariate Cox regression models. For the evaluation of OS, PFS, and LDFS, the Kaplan-Meier (KM) method was used, and survival curves were generated using R software version 4.2.1. Bar graphs were created using PRISM 8.0.

## Results

### Clinical data

**Figure 1:**
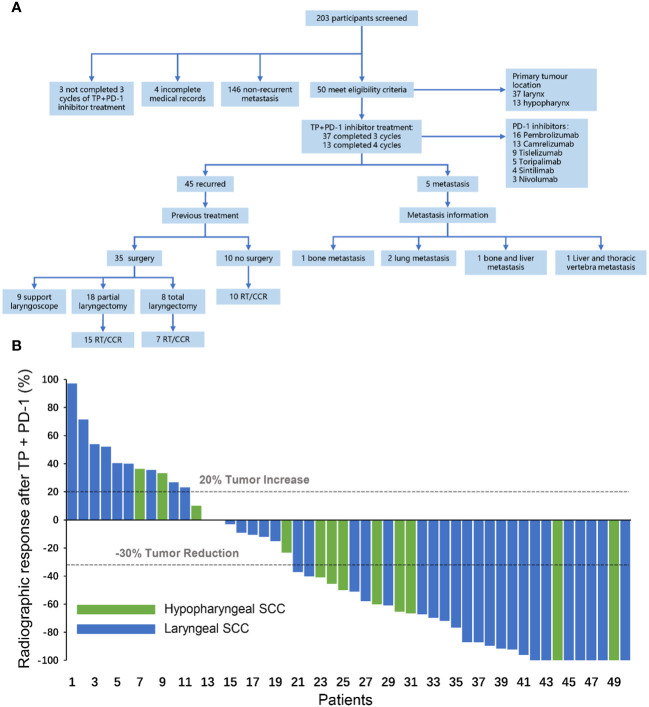
**(A)** Flow chart for inclusion of patients with recurrent and metastatic hypopharyngeal/laryngeal squamous cell carcinoma. Support laryngoscope, CO2 laser hypopharyngeal/laryngeal cancer resection under support laryngoscope; Total laryngectomy, total laryngectomy ± partial hypopharyngectomy/total hypopharyngectomy ± selective neck lymph node dissection; Partial laryngectomy, partial laryngectomy ± partial hypopharyngectomy ± selective neck dissection; RT, radiotherapy; CCR, concurrent chemoradiotherapy. **(B)** Radiographic response after TP+PD-1 inhibitors. CR, complete response; PR, partial response; SD, stable disease; PD, progressive disease; TP, paclitaxel (Albumin-bound)+cisplatin; SCC, squamous cell carcinoma.

A total of 203 patients with RMLSCC/RMHSCC who received TP+PD-1 inhibitor therapy were identified in this study. 153 patients were excluded, including 146 patients without recurrence or metastasis, 4 patients with incomplete medical records, and 3 patients who did not complete 3 cycles of TP+PD-1 inhibitor therapy. Finally, 50 patients with recurrent or metastatic hypopharyngeal/laryngeal cancer who received TP+PD-1 inhibitor therapy were included in this study, comprising 37 cases of laryngeal cancer and 13 cases of hypopharyngeal cancer. Of these, 37 patients completed 3 cycles of TP+PD-1 inhibitor therapy, and 13 patients completed 4 cycles of TP+PD-1 inhibitor therapy. Among the included patients, 45 experienced recurrence, while 5 had distant metastases. Among the recurrent patients, 35 had undergone surgery before TP+PD-1 inhibitor therapy (9 underwent C02 laser surgery under supporting laryngoscope, 18 underwent partial laryngectomy, and 8 underwent total laryngectomy), and 10 patients received concurrent chemoradiotherapy before TP+PD-1 inhibitor therapy. Among the patients with distant metastases, 1 had bone metastasis, 2 had lung metastasis, and 2 had multiple systemic metastases. Regarding the use of PD-1 inhibitors, 13 patients received pembrolizumab, 9 received nivolumab, 4 received cemiplimab, 5 received tislelizumab, 3 received navolimab, and 16 received camrelizumab (**Figure 1A**). General patient characteristics are shown in **Table 1**.

**Table 1:** Baseline Characteristics of the Study Cohort.

Characteristics	Total (n=50)
Sex	
Male	49
Female	1
Primary site	
Larynx	37
Hypopharynx	13
ECOG	
0 or 1	42
2	8
Smoking status	
Never	17
Current or former	33
PD-1 inhibitors	
Pembrolizumab	16
Toripalimab	5
Camrelizumab	13
Tislelizumab	9
Nivolumab	3
Sintilimab	4
T-Stage	
T1-T2	13
T3-T4	37
N-Stage	
N0-N1	24
N2-N3	26
M-Stage	
M0	41
M1	9
Efficacy of TP+PD-1 inhibitor	
CR/PR	28
SD/PD	22
Treatment cycles	
3 cycles	37
4 cycles	13

**Figure 2:**
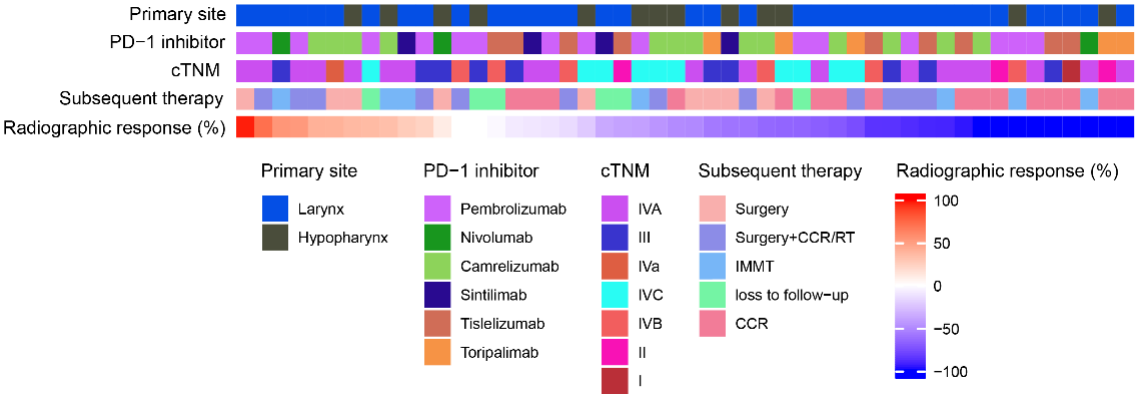
Heat map of patient treatment related information. RT, radiotherapy; CCR, concurrent chemoradiotherapy; IMMT, Immune monotherapy maintenance therapy.

**Figure 2** showed the cTNM staging, PD-1 inhibitors, initial tumor site, and subsequent treatment information for 50 patients, arranged based on the radiographic response. After the 50 patients had undergone 3–4 cycles of TP+PD-1 inhibitor treatment, 21 of them underwent surgery (12 of those patients subsequently received concurrent chemoradiotherapy/radiotherapy), 16 patients received concurrent chemoradiotherapy/radiotherapy, 7 patients received Immune monotherapy maintenance therapy, and 6 patients were lost to follow-up.

### Efficacy evaluation

After completion of TP+PD-1 inhibitor therapy, the ORR in patients was 56.0% (28/50), with 9 patients (18.0%) achieving CR and 19 patients (38.0%) achieving PR. Among the PR patients, 7 (14.0%) had major PR and 12 (24.0%) had minor PR.8 patients (16.0%) had SD, and 14 patients (28.0%) had PD (**Figure 1B**).

### Survival analysis

As of August 15, 2023, the follow-up was completed with a median follow-up time of 16.4 (range: 5.63–41.1) months. Among the 50 patients, 37 were alive and 13 had died. Among the 13 deceased patients, 12 deaths were related to hypopharyngeal cancer, including 10 cases of primary site recurrence or cervical lymph node metastasis and 1 case of distant metastasis (2 cases of lung metastasis). One death was due to non-tumor-related causes, specifically pulmonary infection.

**Figure 3:**
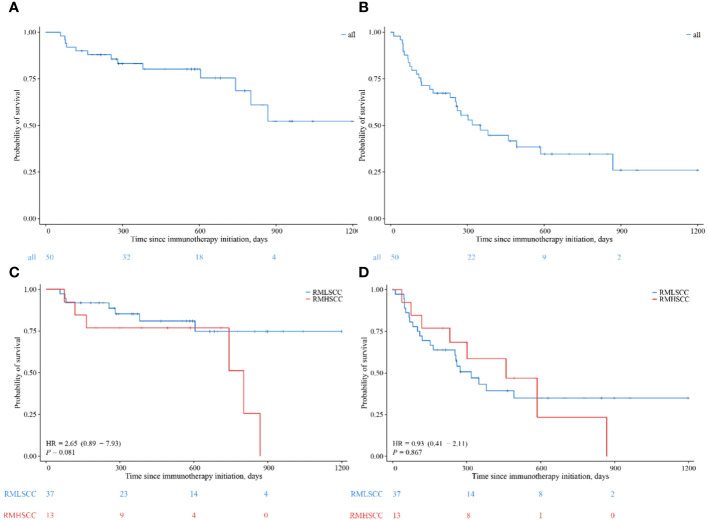
**(A, B)** Overall Survival and Progression-Free Survival in the Entire Cohort. **(C, D)** Kaplan–Meier curves comparing the overall survival **(C)** and the progression-free survival **(D)** of recurrent metastatic laryngeal squamous cell carcinoma and recurrent metastatic hypopharyngeal squamous cell carcinoma.

Kaplan-Meier method was used to plot survival curves and progression-free survival curves for the 50 patients (**Figure 3**), and 1-year and 2-year OS and PFS endpoint efficacy indicators were calculated along with their respective median values, with 95% confidence intervals (CI). The median OS was not reached, with 1-year OS and 2-year OS being 80.2% (95% CI: 69.3%-92.9%) and 68.6% (95% CI: 52.6%-89.5%), respectively. The median PFS was 11.67 months, with 1-year PFS and 2-year PFS being 44.7% (95% CI: 31.9%-62.5%) and 26.0% (95% CI: 12.6%-53.4%), respectively. Subgroup analysis using the Kaplan-Meier method for hypopharyngeal cancer and laryngeal cancer revealed no statistically significant differences between the two groups in terms of OS and PFS (P ≥ 0.05).

Univariate Cox model analysis of OS: Results from analysis at the P=0.1 significance level indicated that Primary site and TP+PD-1 inhibitor efficacy were associated with OS in patients with RMLSCC/RMHSCC. Multivariate Cox model analysis of OS: Two factors that showed statistical significance in the univariate Cox model analysis were included in the multivariate Cox model. The analysis revealed that only TP+PD-1 inhibitor efficacy difference (P=0.002) was an independent risk factor for decreased OS, with a hazard ratio of 10.539 (95% CI: 2.296 - 48.377) (**Table 2**). Univariate Cox model analysis of PFS: Analysis of ten factors including gender, age, Primary site, ECOG, T stage, N stage, M stage, smoking status, PD-1 inhibitor type, and treatment cycles showed no correlation with PFS in patients with RMLSCC/RMHSCC (P≥0.05), except for TP+PD-1 inhibitor efficacy (P<0.001) (**Table 3**).

**Table 2:** Univariate analysis and multivariate analysis for Overall Survival.

Characteristics	Univariate analysis		Multivariate analysis	
	Hazard ratio (95% CI)	P	Hazard ratio (95% CI)	P
Age	0.965 (0.909 - 1.024)	0.236		
Sex				
Male	Reference			
Female	0.000 (0.000 - Inf)	0.998		
Primary site				
Larynx	Reference		Reference	
Hypopharynx	2.652 (0.886 - 7.934)	0.081	3.183 (1.044 - 9.702)	0.042
ECOG				
0 or 1	Reference			
2	0.897 (0.197 - 4.083)	0.888		
Smoking status				
Never	Reference			
Current or former	2.225 (0.484 - 10.236)	0.304		
PD-1 inhibitors				
Pembrolizumab	Reference			
Toripalimab	0.000 (0.000 - Inf)	0.998		
Camrelizumab	0.554 (0.141 - 2.178)	0.398		
Tislelizumab	0.362 (0.042 - 3.115)	0.355		
Nivolumab	0.751 (0.084 - 6.680)	0.797		
Sintilimab	1.062 (0.191 - 5.891)	0.945		
T-Stage				
T1-T2	Reference			
T3-T4	1.084 (0.296 - 3.969)	0.903		
N-Stage				
N0-N1	Reference			
N2-N3	0.668 (0.224 - 1.996)	0.470		
M-Stage				
M0	Reference			
M1	0.958 (0.207 - 4.437)	0.956		
Efficacy of TP+PD-1 inhibitor				
CR/PR	Reference		Reference	
SD/PD	9.479 (2.091 - 42.961)	0.004	10.539 (2.296 - 48.377)	0.002
Treatment cycles				
3 cycles	Reference			
4 cycles	0.186 (0.024 - 1.433)	0.106		

**Table 3:** Univariate analysis for Progression-Free Survival.

Characteristics	Univariate analysis	
	Hazard ratio (95% CI)	P
Age	0.994 (0.951 - 1.040)	0.808
Sex		
Male	Reference	
Female	0.000 (0.000 - Inf)	0.997
Primary site		
Larynx	Reference	
Hypopharynx	0.933 (0.412 - 2.110)	0.867
ECOG		
0 or 1	Reference	
2	1.508 (0.610 - 3.729)	0.374
Smoking status		
Never	Reference	
Current or former	0.806 (0.371 - 1.752)	0.586
PD-1 inhibitors		
Pembrolizumab	Reference	
Toripalimab	0.801 (0.222 - 2.891)	0.735
Camrelizumab	0.555 (0.214 - 1.440)	0.226
Tislelizumab	0.487 (0.135 - 1.755)	0.271
Nivolumab	1.013 (0.218 - 4.711)	0.987
Sintilimab	2.096 (0.559 - 7.859)	0.273
T-Stage		
T1-T2	Reference	
T3-T4	0.881 (0.373 - 2.081)	0.773
N-Stage		
N0-N1	Reference	
N2-N3	1.282 (0.606 - 2.711)	0.516
M-Stage		
M0	Reference	
M1	0.638 (0.221 - 1.840)	0.406
Efficacy of TP+PD-1 inhibitor		
CR/PR	Reference	
SD/PD	7.919 (3.487 - 17.985)	< 0.001
Treatment cycles		
3 cycles	Reference	
4 cycles	0.578 (0.234 - 1.428)	0.235

### Adverse reactions to TP+PD-1 inhibitor therapy

**Table 4** presents the adverse events observed in all 36 patients, categorized by symptom severity (Grade 1–2 and Grade 3–4). The most common adverse events included rash, neutropenia, nausea, vomiting, decreased appetite, and cough. Grade 3–4 adverse events were relatively rare, with dermatitis acneiform, rash, and neutropenia being the most notable. 14 patients experienced skin and subcutaneous tissue disorders, with 5 patients requiring steroid therapy, and two patients needing to interrupt TP+PD-1 treatment (1 case of Dermatitis acneiform and 1 case of rash). 8 patients experienced neutropenia, with 3 requiring recombinant human granulocyte colony-stimulating factor injections and interruption of TP+PD-1 therapy. 7 patients developed hypothyroidism, all of whom were treated with levothyroxine tablets. For additional detailed information, refer to **Table 4**.

**Table 4:** Adverse Events.

All (n=36)			
Symptoms	Grade 1–2	Grade 3–4	Total
Skin and subcutaneous tissue disorders			
Dermatitis acneiform	2	1	3
Rash	10	1	11
Blood and lymphatic system disorders			
Anaemia	3	0	3
Neutropenia	5	3	8
Thrombocytopenia	2	1	3
Gastrointestinal disorders			
Constipation	1	0	1
Diarrhoea	3	0	3
Nausea	5	0	5
Stomatitis	1	0	1
Vomiting	5	0	5
General disorders and administration site conditions			
Asthenia	3	0	3
Fatigue	3	0	3
Mucosal inflammation	1	0	1
Pyrexia	6	0	6
Metabolism and nutrition disorders			
Decreased appetite	8	0	8
Hypokalaemia	2	0	2
Hypomagnesaemia	1	0	1
Respiratory, thoracic and mediastinal disorders			
Cough	6	0	6
Endocrine disorders			
Hypothyroidism	7	0	7

## Discussion

The escalating global incidence of head and neck squamous cell carcinoma (HNSCC) has led to the adoption of advanced treatment methods. Currently, standard care for advanced HNSCC includes surgical resection with adjuvant radiotherapy, sometimes combined with platinum-based chemotherapy, or concurrent chemoradiotherapy. Despite these approaches, patients often face high recurrence and mortality rates. This situation has fueled the exploration of new treatments, notably the use of PD-1 inhibitors in immunotherapy, which are now being investigated for their potential in pre-surgical settings. Our study presents a comprehensive analysis of neoadjuvant chemoimmunotherapy in treating recurrent metastatic laryngeal squamous cell carcinoma (RMLSCC) and recurrent metastatic hypopharyngeal squamous cell carcinoma (RMHSCC). The findings indicate a promising direction in the treatment of these cancers, particularly when considering the historical context of their traditionally poor prognosis.

Various studies have delved into the efficacy of PD-1 inhibitors in the treatment of head and neck squamous cell carcinoma (HNSCC). A meta-analysis revealed that the median overall survival (mOS) for patients receiving PD-1 and PD-L1 inhibitors was 7.97 months, ranging from 6.0 to 16.5 months (12, 13). The median progression-free survival (mPFS) reported across these studies was 2.84 months, with a span of 1.9 to 6.5 months. Additionally, a comprehensive review encompassing 13 studies with 1798 patients showed that overall survival after PD-1/PD-L1 checkpoint inhibition varied from 6 to 13 months. This review underscored a 37% reduction in the risk of death and a 77% decrease in the risk of any-grade treatment-related adverse events (TRAEs) when comparing immunotherapy to standard care (5, 14). In our research, we observed a median progression-free survival (PFS) of 12.7 months and a one-year overall survival (OS) rate of 82.8% (15). Our study demonstrates that patients with RMLSCC and RMHSCC can also benefit from PD-1 inhibitors. Notably, the OS and PFS outcomes in our cohort are comparable to, those observed for PD-1 inhibitors in the broader context of head and neck squamous cell carcinoma.

In our study, we assessed 57 patients who underwent combined treatment with PD-1 inhibitors and chemotherapy. The Objective Response Rate (ORR) observed in this cohort was 59.6%. Among those with available post-treatment imaging data, CR was observed in 21.1% of patients (12 patients), and PR in 38.6% (22 patients). These results highlight the efficacy of the neoadjuvant chemoimmunotherapy approach in treating RMLSCC and RMHSCC. The high ORR, particularly the significant proportion of patients achieving CR, underscores the potential of this treatment modality in not only controlling the disease but also in potentially eradicating detectable cancer in a notable fraction of patients. The PR observed in over a third of the patients also indicates a substantial reduction in tumor size, which can be crucial for improving surgical outcomes and reducing the likelihood of recurrence.

Considering the lack of clinical studies investigating PD-1 inhibitors for the treatment of RMHSCC/RMLSCC, the results of this study were particularly noteworthy because the OS, PFS, and ORR in this study were comparable to, or even better than, previous reports on PD-1 inhibitors for the treatment of R/M HNSCC. These findings confirmed the efficacy of PD-1 inhibitors in treating these specific cancer subtypes.

Our study utilizes a retrospective design to evaluate neoadjuvant chemoimmunotherapy in patients with RMHSCC/RMLSCC. While providing valuable insights, this approach comes with inherent limitations that must be acknowledged in interpreting the results. Firstly, the retrospective nature of the study inherently limits the ability to control for potential confounding factors that may influence outcomes. Unlike prospective studies, retrospective analyses rely on existing records and data, which might not encompass all relevant variables or be as rigorously collected. This can lead to potential biases in the study findings (16). Secondly, the small sample size of our study poses a limitation to the generalizability of the results. While the findings are promising, larger studies are needed to validate these results and ensure that they are representative of the broader patient population. Small sample sizes also limit the statistical power of the study, potentially leading to less reliable estimates of treatment effect. Furthermore, the short follow-up duration in this study is a significant limitation, particularly in understanding the long-term outcomes of the treatment. Cancer treatment efficacy is often best evaluated over extended periods, as this allows for the assessment of long-term survival, recurrence rates, and late-onset side effects. A longer follow-up would provide more comprehensive data on the durability of the treatment response and the overall survival benefits. The CPS score was a comprehensive positive score used to evaluate the intensity of PD-L1 staining, assessing tumor sensitivity to immunotherapy by calculating the ratio of the total number of PD-L1-positive immune cells (including tumor cells, lymphocytes, macrophages, etc.) to the total number of viable tumor cells in the sample. The CPS score was crucial for predicting the efficacy of immunotherapy. However, in this retrospective study, only 16 patients treated with pembrolizumab had CPS scores available, preventing further analysis of CPS scores. Despite these limitations, the study contributes valuable preliminary data on the effectiveness of neoadjuvant chemoimmunotherapy in RMLSCC and RMHSCC (17). It underscores the need for larger, prospective studies with longer follow-up durations to fully ascertain the benefits and risks of this treatment approach. Moreover, the study highlights the potential of PD-1 inhibitors in improving outcomes for these challenging to treat cancers, setting a foundation for future research and clinical trials in this area.

## Conclusion

In conclusion, while our study presents promising preliminary data on the effectiveness of chemoimmunotherapy in treating RMLSCC and RMHSCC, further research is required to fully establish its role in the therapeutic landscape of head and neck cancer. This research paves the way for future clinical trials and investigations, aiming to improve treatment outcomes and quality of life for patients suffering from these aggressive forms of cancer.

## Data availability statement

The raw data supporting the conclusions of this article will be made available by the authors, without undue reservation.

## Ethics statement

The studies involving humans were approved by Ethics Committee of Sun Yat-sen University Cancer Center. The studies were conducted in accordance with the local legislation and institutional requirements. The participants provided their written informed consent to participate in this study.

## Author contributions

QF: Conceptualization, Data curation, Formal Analysis, Resources, Writing – original draft, Writing – review & editing. XDL: Investigation, Writing – original draft, Methodology, Project administration. PX: Project administration, Validation, Writing – review & editing, Conceptualization, Methodology. FC: Methodology, Project administration, Validation, Writing – original draft. DW: Data curation, Formal Analysis, Methodology, Project administration, Supervision, Validation, Writing – original draft, Writing – review & editing. XZ: Conceptualization, Investigation, Software, Writing – original draft. CC: Conceptualization, Project administration, Software, Writing – original draft. JG: Methodology, Project administration, Writing – original draft. YS: Formal Analysis, Investigation, Resources, Writing – review & editing. XKL: Conceptualization, Funding acquisition, Investigation, Methodology, Project administration, Resources, Supervision, Validation, Writing – original draft, Writing – review & editing.
